# Generation of Islet-like Cell Aggregates from Human Adipose Tissue-derived Stem Cells by Lentiviral Overexpression of PDX-1

**Published:** 2015-05-01

**Authors:** M. Bahrebar, M. Soleimani, M. H. Karimi, A. Vahdati, R. Yaghobi

**Affiliations:** 1 *Department of Biology, Science and Research Branch, Islamic Azad University, Fars, Iran*; 2 *Department of Hematology, Faculty of Medical Sciences, Tarbiat Modares University, Tehran, Iran*; 3 *Transplant Research Center, Shiraz University of Medical Sciences, Shiraz, Iran*

**Keywords:** PDX-1, Adipose, Mesenchymal stem cells, Islet

## Abstract

**Background:**

Pancreatic duodenal homeobox1 (PDX-1) is a transcription factor that is important in regulating pancreas development and maintaining β-cell function. β-cell replacement is an effective approach for the treatment of type 1 diabetes. Human adipose-mesenchymal stem cells (hAMSCs) are the ideal population cells for differentiating into insulin-producing cells.

**Objective:**

To determine if islet-like cell aggregates production could be generated from hAMSCs by lentiviral overexpression of PDX-1.

**Methods:**

After isolation of hAMSCs, characteristics of these cells were identified by flow-cytometic analysis and multilineage differentiation studies. PDX-1 gene delivered into hAMSCs through lentiviral vector for differentiating hAMSCs into insulin-producing cells (IPCs) at the utilized protocol for 14 days. Characteristics of IPCs were evaluated by immunocytofluorescence, dithizone staining, and quantitative reverse transcription PCR. In response to high glucose medium, insulin release was detected by chemiluminescence enzyme immunoassay.

**Results:**

The islet-like cell aggregates appeared about 10 days after introduction of PDX-1 into hAMSCs. PDX-1 induced its own expression (auto-induction), a number of islet-related genes such as Ngn3, Nkx2-2, and insulin. The insulin-positive cells were detected in the PDX-1 transduced cells. In response to glucose challenge test, secretion of insulin hormone in the medium with high glucose concentration significantly increased in the PDX-1-transduced cells related to medium with low glucose concentration.

**Conclusion:**

Introduction of lentiviral PDX-1 significantly induces hAMSCs to differentiate into islet-like cell aggregates, which may provide a source of adipose stem cells-derived insulin-producing cells for cell replacement therapy in type 1 diabetes.

## INTRODUCTION

The loss or dysfunction of insulin-producing β-cells in the pancreas leads to diabetes disease. In type 1 diabetes, the insulin-producing cells of pancreatic islet are destroyed by the immune system, whereas type 2 diabetes is caused by a combination of insulin resistance and inadequate insulin secretion. Diabetes is associated with serious long-term complications such as cardiovascular disorders, kidney diseases, and blindness [1]. Recent progress in stem cell biology has raised hopes for the generation of regulated insulin-producing cells by differentiation from various sources of stem/progenitor cells [2]. Although embryonic stem (ES) cells can be induced to differentiate into insulin-producing cells [3], these cells, like induced pluripotent stem cells, may form teratomas after transplantation [4].

The characteristics of mesenchymal stem cells (MSCs) or adult stem cells (*eg*, inherent ability for self-renewal, proliferation, and differentiation toward mature tissues, and depending on the surrounding microenvironment) make them very attractive for use in cell therapy and regenerative medicine [5]. Adult stem cells are found in fat [6], bone marrow (BM) [7], pancreas [8], and other tissue. Recent studies suggest that adipose tissue-, bone marrow-, and pancreatic-derived hMSCs [6-8] have the potential to adopt a pancreatic endocrine phenotype. The adipose-derived MSCs are suggested as an attractive source for β-cell replacemen [9, 10]. The adipose-derived MSCs related to other sources can easily be obtained abundantly and are easily accessible in diabetic or healthy individuals. The MSCs comprise only a minor fraction of BM and other tissues, with bone marrow MSCs (BMMSCs) constituting a mere of 0.0001%–0.01% of all BM-nucleated cells. In contrast, adipose tissues contain 100,000 MSCs in each gram of fat. Further, the differential capacity of adipose tissue-derived MSCs (AMSCs) is less affected by donor age. Adipose tissue is an accessible, abundant, and reliable site for the isolation of adult stem cells suitable for tissue engineering and regenerative medicine applications. In this regard, the treatment efficacy of AMSCs for various diseases has been reported using animal models [10]. These cells could be obtained from a diabetic individual by endoscopic surgery or simple liposuction followed by *in vitro* expansion and differentiation into insulin-producing cells (IPCs) or islet-like cell aggregates (ICAs) for subsequent autologous transplantation [11].

To ameliorate the symptoms of type 1 diabetes, a large number of islet cells should be used for transplantation. This problem could be solved by finding ways to generate more islet cells [12]. In recent years, genetic reprogramming of adult human cells in transcription level is an attractive approach for generating cell-based therapy of degenerative diseases like diabetes [13]. Among the potentially useful transcription factors for the induction of β-cell differentiation from non-β-cells, the pancreatic duodenal homeobox-1 (PDX-1) is the most outstanding [14]. PDX-1, a homeodmain-containing transcription factor was shown to have extensive roles in regulating pancreas development and maintaining β-cell function [15]. The homeodomain transcription factor PDX-1 is expressed in the pancreatic endoderm and essential for its early development and later becomes restricted to β cells. In adult animals, PDX-1 regulates the expression of insulin, Glut-2, and glucokinase genes that these genes play an essential role in the function of β-cells. The role of PDX-1 was demonstrated by showing that mutant mice do not develop any pancreatic tissue. Classic methods of gene transfer, such as transfection, are inefficient and limited mainly to delivery into actively proliferating cells *in vitro*. The development of viral vectors gene delivery system enhanced the efficiency of specified differentiation and obtained the homogenous cell populations. Several kinds of virus, lentivirus, adenovirus, adeno-associated virus, and herpes simplex virus, have been manipulated for use in gene transfer application. The lentiviral vectors have advantages related to other viruses include their inactivation by human complement, ability to stably transducer dividing cells, the inability to express any viral proteins that could be immunogenic the ability to achieve long-term transgene expression (over two years) in humans. Other viruses have some drawbacks including limited duration of transgene expression and immunogenicity *in vivo*. Inductions of pancreatic stem/progenitor cells into insulin-producing cells by adenoviral-mediated gene transfer technology have been reported [17]. However, transduction efficiencies and transgene expression levels in MSCs had been found to be higher with lentiviral vectors than adenoviral vectors [16]. Recently some experiments have been performed with using lentiviral vectors expressing key master transcription factors such as PDX-1 to force differentiation of MSCs into insulin-producing cells [18, 19]. Kim and co-workers showed that human AMSCs were successfully differentiated into insulin-producing cells by PDX-1 gene transfer technology based on lentiviral vector system. Those findings demonstrated that a combination of nicotinamide, B27 and N2 in low glucose medium effectively promotes PDX-1 expressing hAMSCs differentiation into insulin-producing cells [20]. Kushner, *et al*, reported that PDX-1 restored β-cell function in Irs2 knockout mice. Mutation in PDX-1 causes autosomal forms of early-onset diabetes (maturity-onset diabetes of the young type 4 [MODY4]). PDX-1 plays an important role in islet development and β-cell function. It is required for pancreas formation during the initial stages of gut development. In adult mice, PDX-1 promotes the expression of proinsulin, Glut2, and glucokinase, which mediates glucose-sensitive insulin secretion. PDX-1 also regulates expression of FGFs and their receptors, which might promote β-cell growth and proinsulin processing via the prohormone processing enzyme PCI/3. Consequently, disruption of PDX-1 in murine β-cell reduces insulin secretion and causes progressive β-cell loss, which culminates in glucose intolerance and diabetes. Moreover, PDX-1 is required in adult humans to promote normal glucose sensing and insulin secretion, and mutations in PDX-1 represent a risk factor for type 2 diabetes. They suggested that dysregulation of PDX-1 by genetic or functional mechanisms might be a common element in autosomal early-onset (MODY) and common type 2 diabetes [21]. However, only few reports demonstrated robust differentiation of human MSC into adequate insulin-producing cells. The low ratio of insulin producing cells in previous investigations still limits their medical application [22]. Previous studies showed that high glucose concentrations were essential for complete differentiation of PDX-1/PDX1-VP16 lentiviral transduced liver stem cells into insulin-producing cells [23]. Because β-cell replacement is an effective approach for the treatment of type 1 diabetes, the hAMSCs are the ideal candidates for cell replacement therapy, and effective role of high glucose on the stem cells differentiation toward β-cell development, the current study was performed to investigate the effects of PDX-1 alone (without growth factors-containing medium) in the high glucose medium, on the transdifferentiation of the hAMSCs into IPCs.

## MATERIALS AND METHODS

Isolation and Culture of hAMSCs 

Human adipose tissues were obtained by simple liposuction from the abdominal subcutaneous fats, as previously described [9]. Briefly, fat samples taken from healthy individuals were washed and digested in phosphate-buffered saline (PBS) supplemented with 0.2% collagenase-type-II (1 mg/mL) (Sigma-Aldrich, USA) under gentle agitation pre-warmed to 37 °C for 45 min. The collagenase was inactivated with an equal volume of Dulbecco’s modified Eagle’s medium (DMEM) containing 10% (v/v) fetal bovine serum (FBS); the digested tissues were filtered through a 100-µm mesh filter to remove cellular debris and centrifuged at 470 g for 5 min to obtain a pellet. The pellet was resuspended in DMEM containing 10% FBS (Sigma-Aldrich, USA). The cell suspension was recentrifuged at 470 g for 5 min. The supernatant containing mature adipocytes was discarded and the pellet containing stromal vascular fraction was resuspended in expansion medium. Expansion medium contained DMEM with 10% FBS, 2 mM L-glutamine, 100 U/mL penicillin, and 100 µg streptomycin. The cells were plated at density 2×10^5^/cm^2^ into T25 culture flasks at 37 °C, and 5% CO_2_. After 24 h, the cell adhesion was checked under an inverted microscope; the non-adherent cells were removed by washing with PBS. The cell medium was replaced with fresh expansion medium. The study was approved by the Ethics Committee of Shiraz University of Medical Sciences (The study protocol conformed to the ethical guidelines of the 1975 Declaration of Helsinki).

Flow Cytometry Analysis

The hAMSCs were detached from the tissue culture flasks at the third passage and stained with FITC-conjugated mouse anti-human antibodies include CD45 and CD90, PE-conjugated mouse anti-human CD34 and CD105. FITC-conjugated mouse anti-human IgG2 and PE-conjugated mouse anti-human IgG1 were used as isotype (all from BD Pharminogen, CA). Data were analyzed using windows multiple document interface for flow cytometry (Win MDI) software (Scripps, CA). Forward- and side-scatter parameters were used to gate live cells.

*In Vitro* Multilineage Differentiation Studies

Adipogenesis and osteogenesis of hAMSCs were evaluated in the appropriate induction media according to the previously reported methods [10]. To induce adipogenic differentiation, 15×10^3^ hAMSCs after third passage were plated in 4-well culture plates. The cultured cells were treated with adipogenic medium for 3 wk. Adipogenic medium consisted of high glucose-DMEM supplemented with 10% FBS, 100 U/mL penicillin, 100 µg of streptomycin and treated with 1.7 µM insulin, 500 µM isobutylmethylxanthine, 200 µM indomethacin (Sigma-Aldrich, St. Louis, USA) and 1 µM dexamethasone. Adipogenesis was assessed by Oil Red O-staining. For this purpose, the cells was fixed in 10% (v/v) formaldehyde solution in aqueous phosphate buffer. Then, the cells were washed in 60% isopropanol and then stained with a 0.6% (w/v) Oil red O-solution for 2 min at room temperature. This followed by extensive washing with distilled water prior destaining in 100% (v/v) isopropanol for 15 min. For osteogenic differentiation, hAMSCs after third passage were incubated at 15×10^3^ cells/cm^2^ in an osteogenic medium for 21 days. Osteogenic medium consisted of high glucose-DMEM supplemented with 10% FBS, 100 U/mL penicillin, 100 µg of streptomycin and treated with 1 µM dexamethasone (Sigma-Aldrich, St. Louis, USA), 10 µM β-glycerol phosphate (Sigma-Aldrich, St. Louis, USA), 3.7 g/L sodium bicarbonate (Sigma-Aldrich, St. Louis, USA) and 50 µM ascorbate-phosphates (Sigma-Aldrich, St. Louis, USA). Osteogenesis was assessed by Alizarin red staining kit. Under osteogenic conditions, AdT-MSCs expressed genes and proteins associated with an osteoblasts phenotype, including alkaline phosphatase, type 1 collagen, osteopontin, osteonectin, osteocalcin and bone sialo protein. To assess osteogenic differentiation, the cells were fixed with 90% methanol for 10 min at room temperature and identified by specific histochemical staining for calcium, using the Alizarin red staining kit. The stained material was examined with phase-contrast microscopy [24].

Plasmids Packaging for Virus Construction 

To produce lentiviral vectors expressing PDX-1, 3.5×10^6^ HEK293T cell line was plated in 10 mL of DMEM supplemented with 10% FBS without antibiotics and incubated overnight at 37 °C in 5% CO_2_ incubator. PsPAX2 plasmid containing gag/pol packaging genes, pMD2.G plasmid containing VSV-G and pEZ-Lv105-PDX-1 transfer plasmid (Genecopoeia, USA) containing human PDX-1 gene co-transfected into HEK293T cell line. They were transfected into HEK293T cells through calcium phosphate (CaPO_4_) precipitation reaction using Express-In transfection reagent according to the manufacturer’s instructions (Biosystems, USA). In addition, an empty lentiviral vector (LV-null) was constructed by co-transfection of PCDH-CMV-MCS-EF1-cGFP-T2A-Puro (BioCat-GmbH, Germany) as an empty plasmid along with psPAX2, pMD2.G plasmids into HEK293T cell line for producing backbone lentiviral vector (Lv-null) using the same method. The culture supernatant containing lentiviral particles was harvested 48 hours after transfection, and clarified using a 0.45-µm filter. To confirm the transfection in the Lv-null transduced HEK 293T cells, expression of GFP was checked by fluorescent microscope (Nikon, Japan); expression of PDX-1 was assessed by PCR in the Lv-PDX-1 transduced HEK293T cells. 

Transduction of PDX-1 into hAMSCs by Lentivirus

Human AMSCs at passage three were transduced with the medium containing viral particles at 37 °C in 5% CO_2_ incubator. The test group of hAMSCs was infected with LV-PDX-1 while backbone group of hAMSCs were infected with empty lentivirus (LV-null) at a multiplicity of infection (MOI) of 100 in serum free medium (SFM) [16, 20, 25]. Approximately, 1×10^6^ cells per well were seeded in 6-well plates and 3 mL of un-concentrated lentiviral supernatant and 4 µg/mL of polybrene (Sigma-Aldrich, USA) were added. Transduction was performed at 37 °C for 6 hours in SFM. The hAMSC (un-transduced hAMSC) was also used as control group of hAMSC. After 6 hours, the medium was removed and replaced by DMEM/F12 containing 10% FBS, 17.5 mM glucose and 1% BSA. To confirm transduction 48 hours later, the backbone group cells were collected and the expression of GFP was identified by fluorescent microscopic method. The expression rate of GFP reflects the efficiency of infection in the transduction [25]. To obtain pure transduced hAMSCs of test and backbone groups, selection was performed using 1 µg/mL puromycin.

*In Vitro *Differentiation of hAMSCs into IPCs

The transduced hAMSCs were cultured in basic media composed of DMEM/F12 (Gibco, USA) with 10% fetal bovine serum (FBS, Gibco USA), 100 U/mL of penicillin, 100 µg of streptomycin supplemented with 17.5 mM glucose (high glucose) and 1% BSA. The cells of two groups (test and backbone) were counted for initial seeding density of 1×10^6^ cells/well and resuspended in basic media. The cells were plated to ultra-low attachment of 6-well culture plates and grew at 37 °C in 5% CO_2_ incubator. The medium was removed after 48 hours; then, the cultured cells were washed by PBS followed by changing media every 2 days during 14 days of differentiation process. 

RNA Isolation, cDNA Synthesis and Quantitative Real-time PCR 

Total RNA was extracted from duplicate samples of the backbone and test groups using RNX-Plus according to the manufacturer’s instructions (High Pure RNA isolation kit, CinnaGen, Tehran, Iran). Synthesis of cDNA was carried out with MMuLV reverse transcriptase (RT) and random hexamer according to the manufacturer’s instructions (Fermentas, Lithuania). Standard PCR conditions were as follows: two-step procedure of an initial denaturation at 95 °C for 10 min, followed by cycles circulating 15 sec of 95 °C and 60 sec of annealing/extension at 60 °C. The relative expression of pancreatic-specific genes (PDX-1, Ngn3, Nkx2-2, and Insulin) was measured by quantitative real-time PCR in Rotor Gene™ 6000 (Corbett, Australia). One µL of each RT reaction was amplified in a 20-µL PCR assay volume containing 20 mM MgCl_2_, 0.5 µM each primer (Table 1), and 1X SYBR Green Master Mix (Bioneer, South Korea). Samples were incubated in the Rotor Gene™ for an initial denaturation at 94 °C for 10 min, followed by 40 PCR cycles. The level of target gene expression was determined by the comparative Ct method, whereby the target was normalized to B2M as an endogenous reference. Design of primers performed using oligo-primer analysis software ver 7. The primer sequences and the length of the amplified products used for real-time PCR are summarized in Table 1. 

**Table 1:** The list of primers for detection of human pancreas markers

Human genes	Primer sequence	T_m_ (°C)	Products' lenght
PDX1	F: 5'-ATGGATGAAGTCTACCAAAGC-3' R: 5'-CGTGAGATGTACTTGTTGAATG-3'	60	159 bp
Ngn3	F: 5'-AGAGAGCGTGACAGAGGC-3' R: 5'-GCGTCATCCTTTCTACCG-3'	60	182 bp
Nkx2-2	F: 5'-AGTACTCCCTGCACGGTC-3' R: 5'-GTCTCCTTGTCATTGTCCG-3'	60	103 bp
h-Ins	F: 5'-GAACGAGGCTTCTTCTACAC-3' R: 5'-ACAATGCCACGCTTCTG-3'	59	143 bp
B2M	F: 5'-ATGCCTGCCGTGTGAAC-3' R: 5'-ATCTTCAAACCTCCATGATG-3'	60	91 bp

Immunocytofluorescence

The cells of backbone and test groups were washed by PBS and fixed with cold para-formaldehyde 4% for 20 min at 4 °C and then washed by cold PBS twice for 5 min. The cells were permeabilized with 0.1% Triton X-100/PBS (Sigma, Aldrich, USA) for 5 min at room temperature for detection of insulin. The non-specific epitopes of fixed cells were blocked by incubation in goat serum 5% (Sigma, Aldrich, USA) for 45 min at room temperature then removed it. The cells were stained by rat anti-human insulin antibody (1:200) (R&D systems, MAB1417, USA) and diluted in BSA/PBS 0.2% at 4 °C for overnight. The cells were washed 3 times with PBS-Tween 0.1% (Merck, Germany) for 5 min. For detection of primary antibody, a fluorescent-labeled secondary antibody, mouse anti-rat IgG-FITC (eBioscience, USA), was utilized for 1 hr at RT. Then, the cells were washed three times with PBS-Tween 0.1% for 5 min. PBS was added to the wells and kept in dark. For nuclei staining, 4, 6-diamidoino-2-phenylindole (DAPI) (Invitrogen, USA) was used. The cells were visualized and their images were captured with a fluorescence microscope (Nikon TE-2000, Japan).

Dithizone (DTZ) Staining

A DTZ (Merck, Germany) stock solution was prepared by solving 50 mg of DTZ in 5 mL of dimethyl sulfoxide (DMSO) and stored at –15 °C. *In vitro* DTZ staining was performed by adding 10 µL of the stock solution to 3 mL of culture medium in the three wells of the 6-well culture plates of the two groups (backbone and test) on the day 14. The staining solution was filtered through a 0.2-µm nylon filter and then used as the DTZ working solution. The culture dishes were incubated at 37 °C for 15 min in DTZ solution. Then, the dishes were rinsed three times with HBSS, and the clusters were stained with crimson red and examined by a stereomicroscope [26].

Measurement of Glucose-stimulated Insulin Secretion (GSIS)

The media of transduced cells (backbone and test groups) were collected for assaying spontaneous secreted insulin on the 10^th^ and 14^th^ day of differentiation stages as a point of GSIS test. The medium was collected and stored at –20 °C until being assayed. Two different concentrations of glucose (5.5 mM and 25 mM) were utilized for measurement of GSIS test. After the 14^th^ day of differentiation, transduced cells (backbone and test groups) were cultured in low- or high-glucose condition. The cells were incubated for 1 hr in low-glucose DMEM (5.5 mM glucose), and the medium was collected and stored at –20 °C. Next, the cells were washed with PBS and incubated for 1 hr in high-glucose DMEM (25 mM glucose) and the medium was collected and stored at –20 °C. The measurement of glucose-stimulated insulin release was performed by CLIA system according to the manufacturer’s instructions [27]. 

Statistical Analysis

Data were presented as mean±SD. The differences between groups were analyzed by *Student’s t* test for independent samples and one-way ANOVA using SPSS. Band scoring of the RT-PCR analysis was performed using Lab Image ver 3.3.3 software. For gene expression analysis using real-time PCR, data from the Rotor-Gene^®^ Q and other cyclers were evaluated by Rest 2009 software, Qiagen ver 2.0.13, and RotorGene 6000 series software ver 1.7 (Corbett). A p value <0.05 was considered statistically significant. 

## RESULTS

*In Vitro* Growth and Characterization of hAMSCs 

**Figure 1:**
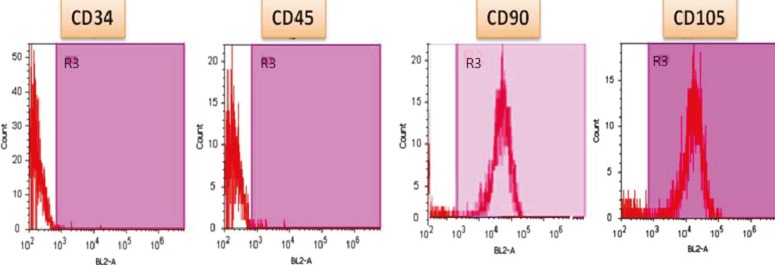
Flow-cytometric analysis of hAMSCs. The cells were positive for CD90 and CD105 and negative for CD34 and CD45 antigens.

**Figure 2:**
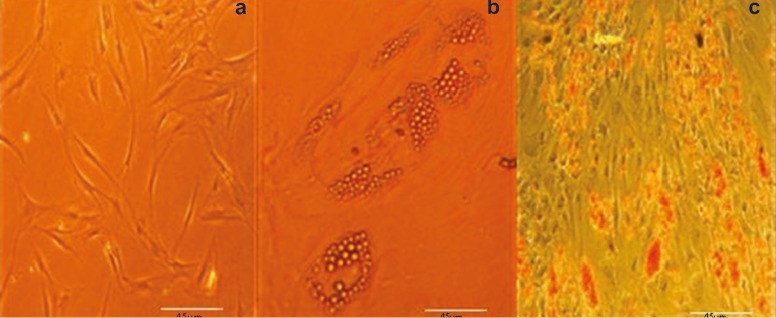
Differentiation of hAMSCs into adipocytes: a) hAMSCs cultured in normal medium served as a control and were negative for this staining (original magnification 100×); b) Non-staining; and c) Oil red O-staining (original magnification 100×). Fat droplets were seen in red on the surface of differentiated cells stained with Oil red, which showed their potential to differentiate into adipocytes

**Figure 3:**
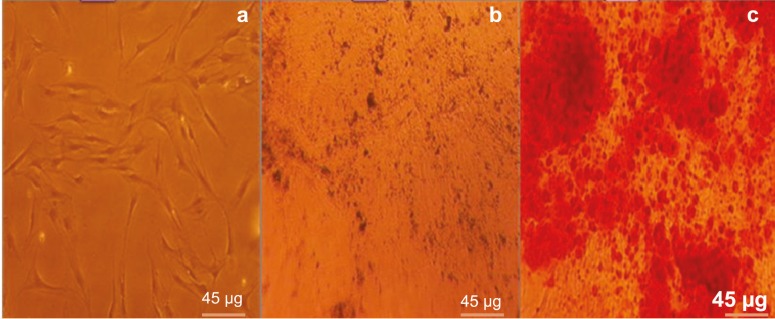
Differentiation of hAMSCs into osteoblasts. a) hAMSCs cultured in normal medium served as a control and were negative for this staining (original magnification 100×); b) Non-staining; and c) Alizarin red staining (original magnification 100×). Red calcium deposits were seen in the cells, which showed their potential to differentiate into osteoblasts

Flow-cytometric analysis of hAMSCs showed that they were positive for CD90 (86.6%) and CD105 (99.8%), and negative for CD34 (0.411%) and CD45 (0.65%) (Fig. 1). Upon specific induction, hAMSCs exhibited *in vitro* competence to differentiate into adipogenic and osteogenic lineages as confirmed by Oil red O-staining (Fig. 2) and Alizarin red staining, respectively (Fig. 3). The initial culture of stromal vascular fraction resulted in the growth of plastic adherent cell population with typical mesenchymal morphology. Because of the adherence character of hAMSCs, non-adherent blood cells removed by medium changes and subsequent passages. After plastic adherence selection, primary hAMSCs exhibited a spindle-shaped fibroblastic morphology for three days (Fig. 2a-3a). Fat droplets were seen in red in the cytoplasm of differentiated cells stained with Oil red, which showed their potential to differentiate into adipocytes. In addition, presence of calcium deposits, characteristic of osteoblasts, in differentiated cells stained with Alizarin red after 21 days of culturing showed their potential to differentiate into osteoblasts and with the notion that hAMSCs posses stem cell properties.

*In Vitro* Differentiation of PDX-1^+^ hAMSCs into IPCs

**Figure 4:**
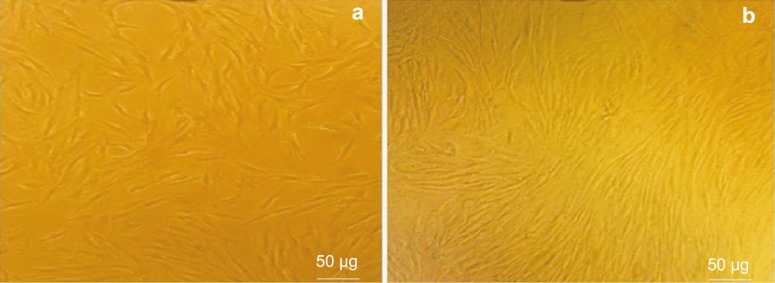
Morphologic changes in the non-treated hAMSCs (Control group). There were no cell clusters formed on a) day 0, and b) day10 (original magnification ×100).

**Figure 5:**
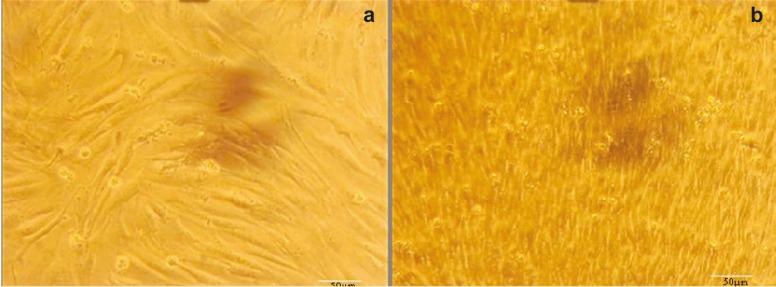
Morphologic changes in the backbone group of hAMSCs (Lv-null- transduced cells). There were no cell clusters formed on a) day 0, and b) day 10 (original magnification 100×).

**Figure 6:**
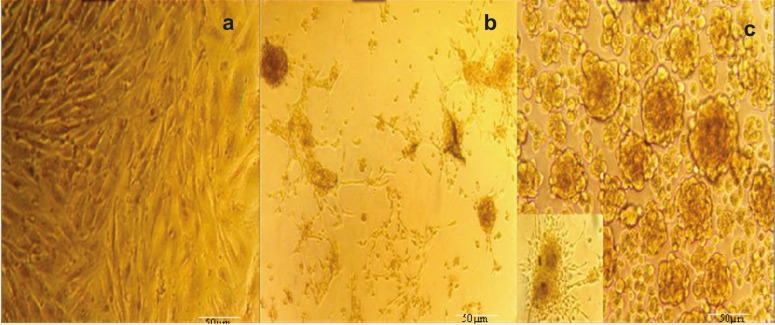
Morphologic changes in the test group (hAMSCs + LvPDX-1). a) Day 0, the cells were spindle shaped and fibroblast-like before induction (original magnification 100×); b) Day 5, the induced cells turned shorter and changed into round epithelial-like cells (original magnification 100×); c) Day10, islet-like cell clusters appeared and large islet-like cell aggregates were observed (original magnification in upper panel 100× in lower panel 400×).

At first day, hAMSCs were spindle-shaped with a fibroblast-like morphology and were attached to the plate during cell culture. These characteristics were well preserved in the control group during repeated subculture for the next 10 days (Fig. 4a-b). In the backbone group, phenotypic changes were similar to non-treated hAMSCs (control group) (Fig. 5a-b). However, in the test group, the cells showed a remarkable transition from bipolar fibroblast-like morphology to a round epithelial-like shape. During further culturing of test group, the rate of cell proliferation became slower and spindle-like cells became short and changed into round epithelial-like cells. Meanwhile, some new islet-like cell aggregates started to appears (Fig. 6b-c). Cellular aggregation occurred as a gradual process and complete aggregates were formed by the 10^th^ day of differentiation (Fig. 6c). Whereas, the control and backbone groups of hAMSCs did not produce any cluster (Fig. 4-5).

Expression Profile of Pancreatic β-cell Development-related Genes 

**Figure 7:**
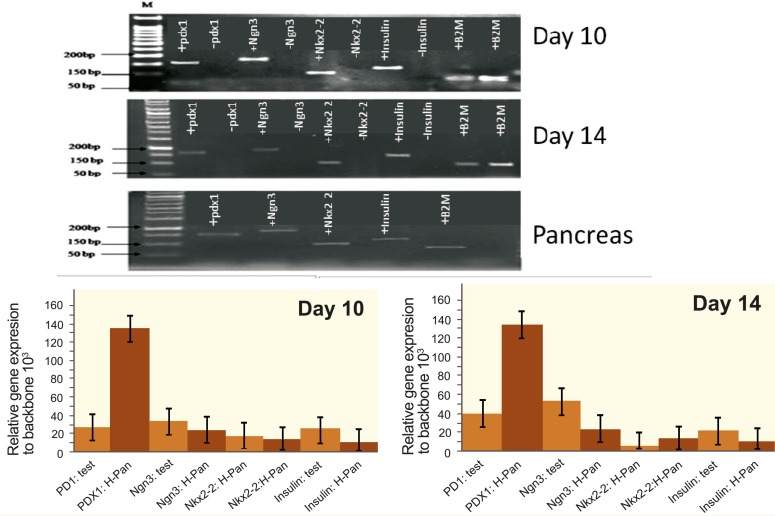
Expression of pancreatic-related genes including PDX-1, Ngn3, Nkx2-2 and insulin with a) RT-PCR or b) real-time PCR at the 10^th^ and 14^th^ day of differentiation. Symbol (+) before each gene name indicates that gene has positive expression in the test group or pancreas tissue sample; symbol (–) indicates that gene has negative expression in the backbone group. Human pancreas tissue was used as a positive control group and B2M gene was used as reference. H-Pan: Human Pancreas

To determine whether the hAMSCs had differentiated into IPCs or ICAs, the expression of genes involved in pancreatic β-cell development like Ngn3, Nkx2-2 and insulin production was examined by qRT-PCR. Endocrine cells differentiation-related genes like PDX-1, Ngn3, Nkx2-2, and insulin were not expressed in backbone group, while these genes in the induced cells of test group expressed at the 10^th^ and 14^th^ day of differentiation (Fig. 7a-b). Relative expression of endocrine cell differentiation-related genes like PDX-1, Ngn3, Nkx2-2, and insulin significantly increased in the PDX-1 transduced cells of the test group in comparative with backbone group (p<0.05), that analyzed by REST 2009 ver 2.0.13 software based on Ct values (Fig. 7 b). The results of relative expression of each gene in the test group in comparison to backbone group are summarized in Table 2. The results showed that relative expression of PDX-1, Ngn3, Nkx2-2, and insulin were significantly increased in the test group in comparison to backbone group (p<0.05).

**Table 2:** The mean relative gene expression in the test group compared to backbone group (sham) on the 10th and 14th day of differentiation

Human genes	Relative gene expression in test group in comparison to backbone group		p value
	Day 10	Day 14	
PDX1	26708.290	40622.742	<0.001
Ngn3	33923.528	53974.861	<0.001
Nkx2-2	16961.781	5853.160	<0.001
Insulin	25006.231	22381.204	<0.001

Immunocytofluorescence for Insulin 

**Figure 8:**
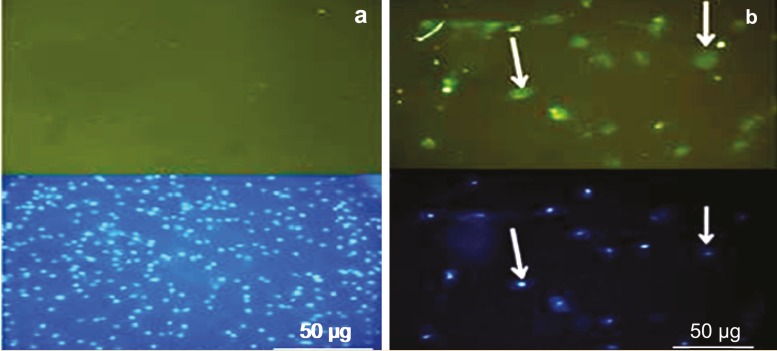
Immunocytofluorescence has been performed to detect insulin-positive cells. a) The negative cells for insulin in backbone group (100×); b) The positive cells for insulin in the test group (400×) that observed with green color in fluorescent microscopic images in the upper parts of the figure (anti-insulin green staining). DAPI-stained cell nuclei were observed in lower parts of the figure for a) backbone group and b) test group. Images were captured using a fluorescent microscope (Nikon, Japan).

To determine insulin production by PDX-1 transduced hAMSCs *in vitro*, immunocytofluorescence was performed on day 14. Insulin immunoreactivity within backbone group revealed negative cells for insulin (Fig. 8a), while the cells of test group were positive for insulin (Fig. 8b). After washing with PBS, cells were incubated with DAPI for cell nuclei staining. The cells were visualized and photomicrographed using a fluorescence microscope. Diffuse immunoreactivity was observed in almost all of the areas of the differentiated cells where stained with anti-human insulin.

Human AMSCs Differentiation and DTZ-staining on Day 14

**Figure 9:**
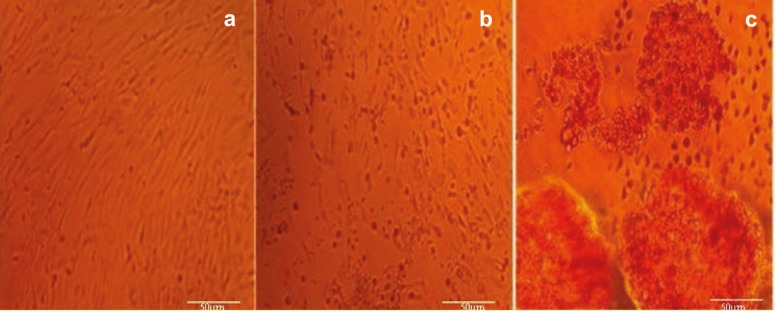
Identification of insulin-producing cells derived from hAMSCs by DTZ staining at day 14 of differentiation. a) Control group was negative for DTZ-staining (original magnification 100×); b) Backbone group was negative for DTZ-staining (origina magnification 100×); c) Most cells of test group were positive for DTZ-staining on day 14 and found that most were crimson red (origina magnification 400×).

To determine whether the hAMSCs differentiated into insulin-producing cells, the control, backbone and test groups of the cells were stained with DTZ. The undifferentiated cells of the control (Fig. 9a) and backbone (Fig. 9b) groups were negative for DTZ-staining. The differentiated cells of test group were stained with DTZ (Fig. 9c). Most of the cells in test group were positive for DTZ-staining on day 14 and were crimson red. Results showed that the insulin-producing cells or islet-like cell aggregations of the test group became fully differentiated and matured on the day 14 of differentiation.

Spontaneous and Glucose-stimulated Insulin Secretion

**Figure 10:**
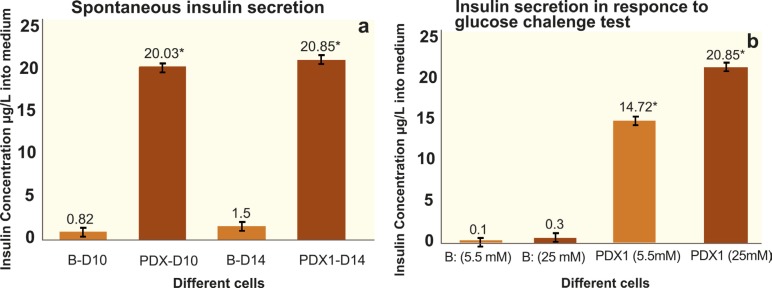
Insulin secretion into medium. a) Spontaneous insulin secretion on the 10^th^ and 14^th^ days of differentiation. b) Insulin secretion in response to glucose challenge test at the 14^th^ day of differentiation. B-D10: Backbone group/hAMSCs + Lv-null on day 10 of protocol; PDX1-D10, test group/hAMSCs + LvPDX-1 on the 10^th^ day of differentiation; B-D14, Backbone group/hAMSCs + Lv-null at day 14 of protocol; PDX1 D14,Test group/hAMSCs + LvPDX-1 on day 14 of differentiation

During the stages of differentiation at the 10^th^ and 14^th^ day, the amount of spontaneous insulin secretion in medium was measured by chemiluminescence enzyme immunoassay method (Fig. 10a). The mean amount of insulin release in the backbone group at days 10 and 14 of differentiation was 0.82 µg/L (19.93 µIU/mL) and 1.50 µg/L (33 µIU/mL), respectively (Fig 10). These amounts did not show significant difference, while the amount of insulin secretion in the test groups of differentiated cells on the 10^th^ and 14^th^ days of differentiation was 20.03 µg/L (480.5 µIU/mL) and 20.85 µg/L (500 µIU/mL), respectively. The test group data showed a significant increase in comparison to the backbone group (p<0.05). Data analysis indicated that insulin release is not significantly different between the test groups. The secretion of insulin was several-fold increase in LvPDX-1 differentiated cells of test group compared to the undifferentiated cells of backbone group on the 10^th^ and 14^th^ days of differentiation. To find whether these insulin-producing cells were responsive to glucose challenge, the amount of insulin secreted by the cells of test and backbone groups in response to different glucose concentrations of medium was measured (Fig. 10b). At day 14 of differentiation, the cells were treated with low (5.5 mM) and high glucose (25 mM) medium. Supernatant were collected and analyzed for insulin release by chemiluminescence enzyme immunoassay method (CLIA). The mean mount of secreted insulin in the medium of test group was 14.72 µg/L (353 µIU/mL) at low glucose and 20.85 µg/L (500 µIU/mL) at high glucose medium that was significantly different (p<0.05) (Fig. 10b). The mean of amounts of secreted insulin in the medium of backbone group was 0.10 µg/L (2.45 µIU/mL) at low, and 0.3 µg/L (2.85 µIU/mL) at high glucose concentration (p>0.05). Results indicated that the release of insulin in the test group at the low and high glucose medium had a significant difference related to backbone groups (p<0.05) (Fig. 10b). 

## DISCUSSION

In this study, the potential of hAMSCs for differentiating into functional insulin-producing cells was assessed after transduced with lentiviral PDX-1. Human hAMSCs exhibited their fibroblast-like morphology and were positive for mesenchymal cell marker (CD90 and CD105) and negative for hematopoietic cell markers (CD34 and CD45). Ability of multilinages differentiation of hAMSCs into adipocytes and osteoblasts were detected by Oil red O and Alizarin red staining. We found that introduction of PDX-1 stimulated the initial process of cell aggregation and cluster formation, an important step in pancreas development and differentiation [28]. The cells showed a remarkable transition from bipolar fibroblast-like morphology to a round epithelial-like shape. Then, these cells became clusters in the medium. These findings were consistent with previous experiments of Boroujeni, *et al*. who showed that transduced hADSCs + PDX-1 became round and clusters in the differentiation medium [19]. These results were further confirmed by immune- and DTZ-staining, gene expression analysis and insulin hormone assays. Examination of the pancreas-related genes expression was carried out by RT-PCR and real-time PCR. 

Our results indicated that ectopic PDX-1 could induce its own expression (auto-induction) as well as some other genes (Ngn3 and Nkx2-2) that induced clusters forming. These results were in accordance with previous findings who showed that the pancreatic progenitor-specific transcription factors such as PDX-1, Ngn3, and Nkx2-2 were expressed in most of the formed clusters and that these genes involved in β-cell development in mice and human [29, 30]. In addition, another researcher showed over-expression of PDX-1 in the transduced hADSCs + PDX-1 followed by significant expression of Ngn3, glucagon, Glut-2 and somatostatin in low glucose medium. It was also shown that insulin-producing cells were produced by non-integrated lentiviral vector harboring PDX-1 gene [19]. Our experiments showed that overexpression of PDX-1 induced overexpression of Ngn3 in high glucose medium. Ngn3 is a bHLH transcription factor that binds to E-box and is involved in endocrine pancreas development [31]. Previous studies demonstrated that PDX-1 is upstream of Ngn3 during embryonic development and PDX-1 expressing progenitors give rise to both exocrine and endocrine lineages, while the Ngn3-expressing progenitors selectively populate the endocrine [32, 33]. These findings showed that differentiation of endocrine progenitors by PDX-1 could be directly affected by Ngn3 regulation or by coordination of PDX-1 with other transcription factors. The role of Ngn3 in giving rise to hormone-expressing cells during the secondary transition depends on PDX-1 [32]. Genetic lineage-tracing studies and the phenotype of Ngn3-null mice demonstrated that all islet hormone-producing endocrine cell types (α, β, δ, ε, and pancreatic polypeptide) derive from Ngn3-expressing progenitors. Transgenic mice overexpressing Ngn3 early in their development, showed a marked increase in endocrine cell formation, which indicates that Ngn3 induces islet cell precursors differentiation [34]. In contrast, mice with targeted disruption of Ngn3 have no endocrine cells [35]. These results suggested that after treatment with LV-105-PDX-1, hAMSCs test group, expressed pancreatic endocrine related factors such as Ngn3 that lead to differentiate into pancreas endocrine lineage. In addition, the factor Nkx2-2 was upregulated in the test group after PDX-1 overexpression. It has been shown that PDX-1 functions in concert with other transcription factors (*eg*, Nkx2-2) to regulate the expression of insulin and several other islet-specific genes. The Nkx2-2, a NK homeoboxe gene, appears at E9.5 in completely pancreatic bud and expressed in the islet cells except in δ cells. It may be required for terminal differentiation of β cells or for the expression of the insulin gene. It is also essential for maintaining of Nk6-1 expression. Nkx6-1 is expressed at E10.5 in the pancreatic bud and is only seen in differentiated β cells and required for expansion and terminal differentiation of β-cell progenitors. Nkx2-2 mutant mice became diabetic after birth and died due to the hyperglycemia. Targeted disruption of Nkx6-1 gene in mice resulted in inhibition of β-cell formation [36]. Nkx2-2 may also be important to preserve mature β-cell function, as in transgenic mice with a repressor form of Nkx2-2. It was shown that Nkx2-2 was expressed in β cells exhibiting reduced levels of GLUT-2 and insulin, and islets displayed a disrupted architecture. Previous findings demonstrated that Nkx2-2 could function as activator of gene transcription in mature β cell. Through binding sites in the promoter or indirectly by its ability to activate MafA, Nkx2-2 may influence the insulin gene expression [37]. According to a previous study, insulin mRNA expression was detected after Ad-PDX-1 introduction alone, while not detected in the backbone group [38].

To evaluate the performance of insulin secreting cells, insulin secretion was detected by ICC, DTZ-staining and chemiluminescence immunoassay method. The ICC analysis revealed diffuse immunoreactivity in almost all of the areas of the induced cells stained with anti-human insulin. DTZ-positive cells became visible on the day 14 of differentiation. ICAs were distinctly stained crimson red by DTZ. DTZ is a zinc-binding substance and pancreatic islets from some species including mouse, dog, pig and human are known to be stained by crimson red treatment, because of their higher zinc contents compared to other tissues. Zinc is required in pancreatic β cells for packaging insulin [26]. Based on these findings, hAMSCs-derived DTZ-stained clusters are suggested to contain pancreatic endocrine cells such as insulin-producing β cells. CLIA analysis demonstrated an ability of test group cells to insulin synthesis and secretion into the media. However, there was an additional increase in the level of medium insulin in response to glucose challenge in differentiated cells of test group, whereas no increase in undifferentiated cells of backbone group was seen. The cells of test group could secreted insulin in a glucose-regulated manner and suggested a dose-dependent response. These results indicate that some glucose-mediated regulation of insulin secretion is achieved by LV-105-PDX-1. In the adults, PDX-1 expression was shown to be largely limited to β cells and to low number of δ cells of the pancreatic islets [39]. Previous research showed that PDX-1 regulates the expression of the Glucose Transporter-2 (GLUT-2) and the glucokinase enzyme in a dose-dependent manner [40]. β cells express these two key proteins to control glucose utilization and sensing. Glucose enters the β cells through GLUT-2-2 and is phosphorylated by glucokinase, which catalyzes the rate-limiting reaction of glucose metabolism. Both GLUT-2 and glucokinase have been shown to be required for β cells to maintain a normal glucose response [40]. Lin and co-workers reported the derivation of insulin-producing cells from human or rat AMSCs by overexpression of PDX-1. Results of their study showed that transduced AMSCs expressed insulin, glucagon, and NeuroD gene which were upregulated following PDX-1 overexpression. They showed that the transduced cells secreted increasing amount of insulin in response to increasing concentration of glucose. Transplantation of these cells under the renal capsule of streptozotocin-induced diabetic rats resulted in lowered blood glucose, higher glucose tolerance, smoother fur, and less contact. Histological examination showed that the transplanted cells formed tissue-like structures and expressed insulin [18, 41]. Glucose-stimulated insulin secretion from pancreatic β cells is regulated by a series of electrogenic events leading to exocytosis of insulin-containing granules. The K_ATP_ channel-dependent mechanism for glucose-stimulated insulin secretion has been established as an important component of stimulus-secretion coupling within the last years [42]. Rises in circulating glucose concentration depolarizes the beta cell by the closing K_ATP_ channels, which results in membrane depolarization, opening voltage-dependent Ca^2+^ channels (VDCCs) and allowing a rise in the intracellular Ca^2+^ concentration that is the main trigger for insulin secretion [25, 42]. 

Zaldumbide, *et al*, showed that PDX-1 bound to sequences within the insulin promoter containing a box in glucose-responsive manner. They showed that PDX-1, Ngn3 and Maf-A expression transactivated the human insulin promoter [43]. In accordance with earlier reports, we found that PDX-1 overexpression markedly stimulated gene expression of Ngn3, Nkx2-2 and insulin in PDX-1 transduced cells in glucose-responsive manner. Secretion of insulin into high glucose medium (17.5 mM) in spontaneous and response to glucose challenge test (25 mM) had the same amounts (Fig. 10). This finding demonstrated that glucose with high concentration (17.5 mM or 25 mM) similarly influenced the responsibility and maturation of IPCs derived from PDX-1 transduced cells. According to previous findings that showed glucose influenced on the activation of PDX-1 for binding to DNA rapidly (within 10–15 min) and involved in the phosphorylation of transcription factors [39, 42], it could be suggested that the glucose influenced the activation of ectopic PDX-1 for binding DNA and its involvement in the activation of transcription factors such as Ngn3 and β cell functional gene such as Glut-2 and insulin in the PDX-1 transduced cells. 

Most of the current clinical gene therapy trials worldwide involve viral vectors derived from retroviruses (including lentiviruses), adenoviruses, adeno-associated viruses, herpes viruses and poxviruses. Further studies are needed to refine their safety, improve targeting of gene transfer, and to provide efficient expression of the desired transgenes. Nonetheless, a solution to this problem was developed by splitting the genomes of the helper plasmid into multiple plasmids so that multiple recombination events would be required to form a replicative competent virus [44]. There is still concern with using lentiviral vectors for safety reasons. One concern involves the possibility that the lentivirus viruses could self-replicate and could be produced during manufacture of the vector in the packaging cell line or in the target cells by a process of recombination. Also, scientists have shown that lentiviruses, such as HIV, are successful and efficient gene delivery vehicles. The field has now turned its attention to producing vectors with built-in safety features to prevent the development of replication competent lentiviruses (RCL). However, even the earliest studies with HIV lentiviral vectors did not generate RCL *in vitro* or *in vivo* [45], but precautions are still very important. More work is needed to ensure safety and transduction efficiency with lentiviral vectors.

In conclusion, we concluded that introduction of lentiviral PDX-1 into human adipose tissue-derived mesenchymal stem cells markedly induced the expression of the pancreatic marker genes such as PDX-1, Ngn3, Nkx2-2 and insulin in these cells with a condition of high glucose medium to differentiate into islet-like cell aggregates. Human adipose tissue-derived mesenchymal cells are easily available and autologous in origin, which may provide a source of adipose stem cells-derived insulin producing cells for cell replacement therapy in type 1 diabetes in the future. 
